# BA-j as a novel CDK1 inhibitor selectively induces apoptosis in cancer cells by regulating ROS

**DOI:** 10.1038/srep13626

**Published:** 2015-09-02

**Authors:** Shixuan Zhang, Yongming Bao, Xiulan Ju, Kangjian Li, Haiyan Shang, Lisha Ha, Yuan Qian, Liang Zou, Xiaodan Sun, Jing Li, Qianru Wang, Qingyu Fan

**Affiliations:** 1 State Key Laboratory of Fine Chemicals, School of Pharmaceutical Science and Technology, Dalian University of Technology, Dalian, Liaoning, China; 2 School of Bioscience and Technology, Dalian University of Technology, Dalian, Liaoning, China; 3 College of Vocational and Technical, Dalian University, Dalian, Liaoning, China

## Abstract

Cyclin-dependent kinase 1 (CDK1) is the only necessary CDK in cell proliferation and a novel target in the development of anticancer drugs. 8-Hydroxypiperidinemethyl-baicalein (BA-j) is a novel selective CDK1 inhibitor with broad spectrum anti-cancer activity (IC_50_ 12.3 μM) and 2 tumor xenografts. Because of the differential mechanisms controlling redox-states in normal and cancer cells, BA-j can capture oxygen free radicals (^·^O_2_^−^) and selectively increase the level of H_2_O_2_ in cancer cells, thereby specifically oxidize and activate the intrinsic apoptosis pathway bypassing the extrinsic death receptor pathway, thus inducing apoptosis in cancer cells rather than in normal cells. BA-j is different from cytotoxic anticancer drugs which can activate both the intrinsic apoptosis pathway and the extrinsic death receptor pathway, and therefore harm normal cells while killing cancer cells. The molecular and biochemical mechanisms of reactive oxygen species (ROS) regulation suggest that BA-j may be developed into a novel anticancer agent.

The discovery of novel anticancer drugs that can selectively induce apoptosis in cancer cells is a challenging and significant task in pharmaceutical science. Cyclin-dependent protein kinases (CDKs) are key signaling molecules in the regulation of the cell cycle. CDKs are specific serine/threonine protein kinases in the cytoplasm and nucleus that act as mediators in signal transduction pathways. CDK1 is the only necessary CDK in cell proliferation, and a novel target in the development of anticancer drugs^1^,^2^,^3^. Recently, the global anticancer drug research community has turned its attention to CDK inhibitors, 20 of which have entered clinical trials^1^,^4^,^5^. However, the selectivity of most of the CDK inhibitors currently in clinical trials is unsatisfactory. Some showed inhibitory activity on CDK2 (i.e., acts on S phase and increases toxicity) and certain side effects because of their complex chemical structures. CDK inhibitors based on organic amine derivatives of flavonoid, such as Flavopiridol^6^,^7^ and P276-00^8^,^9^,^10^, have attracted the most interest. However, because of their poor solubility and bioavailability, low blood concentration, difficulty in catabolism and rapid excretion by glucuronidation, the druggability of these molecules is unsatisfactory.

The most artificially cultivated medicinal species in China is *Scutellaria baicalensis*, a perennial herb whose dried root is commonly used in traditional Chinese medicine for the treatment of hyperlipomia, hypertension, hyperglycemia, inflammation, allergic reactions, viral infections, cancer and so on^11^. The major active ingredients in *Scutellaria baicalensis* are flavonoids and more than 40 flavonoid structures have been identified in this plant^12^,^13^. The most common flavonoid in *Scutellaria baicalensis* is Baicalin (9–21%), and its hydrolyzate, Baicalein (BA), possesses stronger potency. Natural flavonoids are selective CDK1 inhibitors, and BA is the most potent among them with the anti-proliferative activity IC_50_ 25–75 μM^14^,^15^,^16^,^17^,^18^,^19^,^20^,^21^,^22^,^23^. Because of the differential mechanisms controlling redox-states in normal and cancer cells, by regulating reactive oxygen species (ROS) of BA^24^,^25^,^26^,^27^,^28^,^29^,^30^,^31^, ROS can specifically oxidize some enzymes with active site of cysteine. Such as, BA can inhibit CDK1 by oxidizing CDC25C, thus suppressing proliferation in cancer cells^15^,^19^,^25^,^32^. Further, BA can activate the intrinsic apoptotic pathways by oxidizing caspases^15^,^16^,^19^,^21^,^22^,^24^,^25^,^26^,^27^,^28^,^29^,^30^,^33^, bypassing the extrinsic death receptor pathway^16^,^24^,^31^, thus inducing apoptosis in cancer cells and activated lymphocytes rather than in normal cells^17^,^21^,^23^,^30^,^31^,^33^,^34^,^35^. However, the exact biochemical mechanism of BA by the regulation of ROS is only partially understood and so is the way BA regulating ROS.

Oral Baicalin can not be directly absorbed until it has been hydrolyzed into BA by intestinal microflora, yet “enterohepatic efflux effects” inactivate and excrete 95% of BA via glucuronidation and sulfation. Therefore, the level of BA in blood is very low (Cmax 0.26 μM) with poor bioavailability by oral BA^36^,^37^,^38^,^39^,^40^,^41^. In addition, BA is easily oxidized and practically insoluble in water, making it difficult to administer intravenously. Because of its poor bioavailability and undesirable traits as a drug, BA does not meet the requirements for the clinical treatment of cancer^24^.

Therefore, attempts have been made to increase the effectiveness of BA by structural modifications. The most effective structural modifications are likely to be BA Mannich base derivatives^42^,^43^,^44^. In our previous work, dozens of natural flavonoids were used as lead compounds to create hundreds of Mannich base derivatives of flavonoids. Using CDK1/Cyclin B inhibitory activity screening and structure-activity relationship studies, 8-hydroxypiperidine-methyl-baicalein (BA-j) was identified as the most effective flavonoid Mannich base derivative^45^. BA-j is a selective CDK1 inhibitor with a novel chemical structure^45^.

In this paper, the molecular and biological mechanism of BA-j specifically inducing apoptosis in cancer cells was studied and the way BA-j regulating ROS was explored by using a PF1 fluorescent probe to selectively determine the level of intracellular H_2_O_2_. These data provide evidence that BA-j could be developed into a novel anticancer agent for clinical use.

## Results

### Preparation and characterization of BA-j

**Figure 1:**
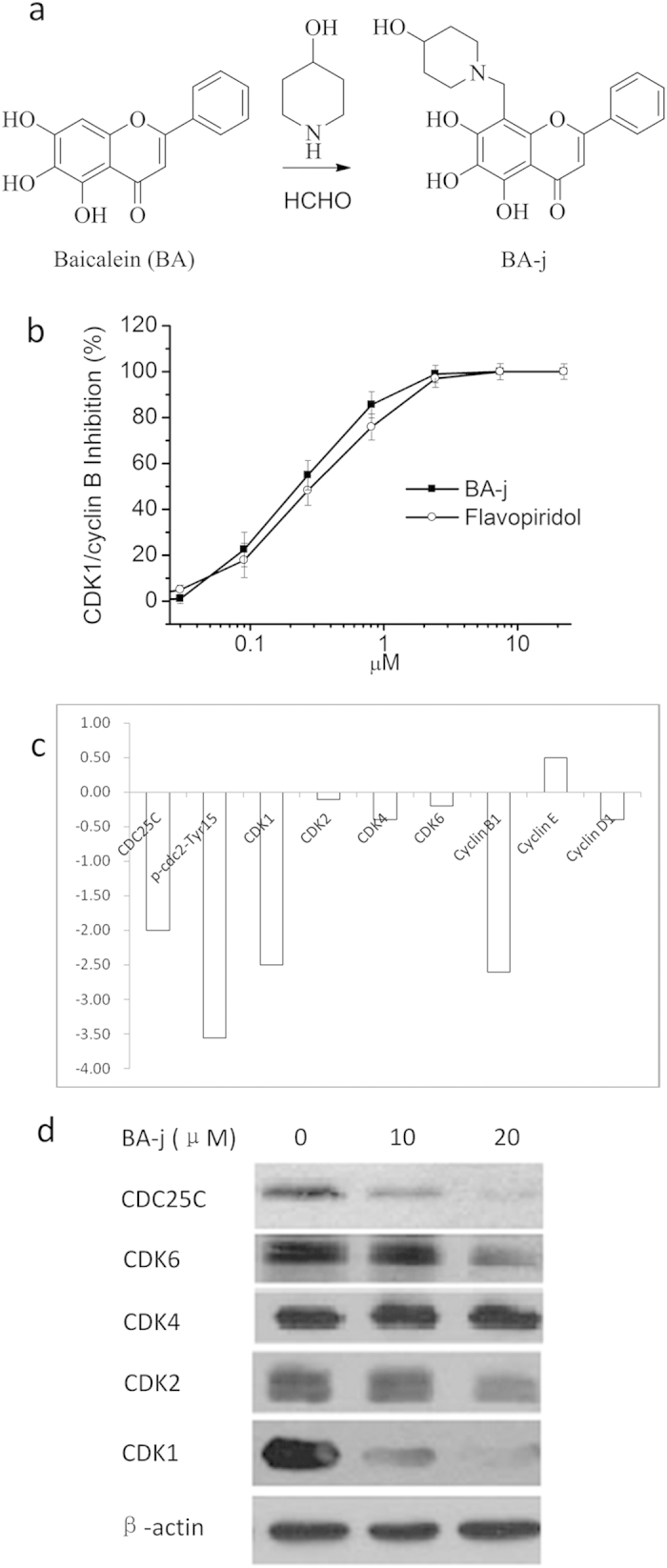
Preparation and Targets screening of 8-Hydroxypiperidinemethyl-baicalein (BA-j). (**a**) Preparation of BA-j. (**b**) Inhibitory activity against CDK1 of 8-Hydroxypiperidinemethyl-baicalein (BA-j). (**c**) Hep G2 cells were treated with 10 μM BA-j for 24 h, and the assay of protein level in HepG2 cell signaling pathways were determined by Western blot for conjugated secondary antibody (goat-antimouse IgG/FITC) using densitometric scanning and normalized using internal standards (β-actin). (**d**) Hep G2 cells were treated with 10 and 20 μM BA-j for 24 h, and the expressions of CDKs and CDC25C proteins in HepG2 cell signaling pathways were determined by Western blot for the appropriate secondary antibody (horseradish peroxidase-conjugated goat-antimouse IgG).

The major active ingredient in the root of *Scutellaria baicalensis* Georgi is Baicalin. Baicalein (BA, Sigma, 98% pure) hydrolyzed from Baicalin was used as a lead compound and reacted with 4-hydroxy-piperidin and formaldehyde in methanol by Mannich reaction to obtain BA-j, with >90% yield, 99.0% purity and <2.0% total related substances (Fig. 1a)^45^.

BA-j is a yellow, solid, crystalline powder, containing 99.1% 5,6,7-Trihydroxy-8-(4-hydroxy-piperidin-1-ylmethyl)-2-phenyl-chromen. Dried under reduced pressure at 80 °C for 4 h, a single melting endothermic peak was shown at 215 °C by DSC. UV (methanol): λmax 277 nm, 322 nm. HRMS (TOF ES^+^) m/z: 384.1441[M+H]^+^ (theoretical value 384.1447), molecular weight (C_21_H_21_NO_6_): 383.14. IR (KBr, cm^−1^): 3059 (ν_ArC-H_), 2961, 2822, 1679 (ν_C=O_), 1574 (ν_ArC=C_), 1549, 770, 696. BA-j CH_3_SO_3_H, ^13^C-NMR (DMSO-*d*_6_, 400 MHz) δ: 182.24 (C-4), 163.25 (C-2), 153.62 (C-9), 149.31 (C-7), 148.34 (C-5), 132.07 (C-1′), 130.81 (C-4′), 129.27 (C-3′,5′), 128.83 (C-6), 126.69 (C-2′,6′), 104.91 (C-8), 104.08 (C-10), 95.90 (C-3), 50.72 (C-4″), 47.60 (C-2″, 6″), 39.50 (C-1″, CH_3_SO_3_H), 29.35 (C-3″,5″). ^1^H-NMR (DMSO-d6, 400 MHz) δ: 8.17-8.15 (m, 2H, Ar-2′, 6′-H), 7.65-7.59 (m, 3H, Ar-3′,4′,5′-H), 7.09 (s, 1H, 3-H), 4.98 (s,1H, 4″-OH), 4.51 (s, 2H, C_8_-CH_2_), 3.67 (m, 1H, 4″-CH), 3.41-3.40 (m, 4H, 2″, 6″-CH_2_), 2.30 (s, 3H, CH_3_SO_3_H), 1.85-1.59 (m, 4H, 3″, 5″-CH_2_). The characteristic peak of BA C_8_-H at δ6.60 disappears and is replaced by a BA-j C_8_-CH_2_ substitute characteristic peak at δ4.17. BA-j is 5, 6, 7-Trihydroxy- 8-(4-hydroxy-piperidin-1- ylmethyl)-2- phenyl-chromen.

The pKa of BA-j is 5.56, logP 0.68 (Octanol/Water), and its solubility is 58 μM in water and 376 μM in PBS. BA-j is stable in PBS in dark conditions. However, at room temperature under light, BA-j can capture. ^·^O_2_^−^, release H_2_O_2_, and be oxidized and hydrolyzed into 8-hydroxymethyl-5, 6-dehydrogenation-baicalein.

### Targets of BA-j screening

Using natural flavonoids as lead compounds, hundreds of flavonoid Mannich base derivatives were semi-synthesized. These derivatives were screened for their inhibitory activity against CDK1/Cyclin B by FRET, and their structure-activity relationship analyzed^44^,^45^. The inhibitory activity of BA-j was the strongest (CC_50_ 0.30 ± 0.11 μM), similar to Flavopiridol (CC_50_ 0.33 ± 0.14 μM), and approximately 20-fold stronger than the lead compound BA (CC_50_ 6.53 ± 0.21 μM), and approximately 50-fold stronger than Baicalin (CC_50_ 14.36 ± 0.23 μM) (Fig. 1b). These results suggest that BA-j as a substrate compound for ATP can directly inhibit the activity of CDK1/ cyclin B.

To confirm the target of BA-j, Hep G2 cells were treated with 10 and 20 μM BA-j for 24 h, then the protein levels of signaling pathways in HepG2 cell were determined by Western blot for conjugated secondary antibody (goat-antimouse IgG/FITC) using densitometric scanning and normalized using internal standards (β-actin) , as shown in Fig. 1c. Among them, the expressions of CDKs and CDC25C proteins in HepG2 cell were determined by Western blot for the appropriate secondary antibody (horseradish peroxidase-conjugated goat-antimouse IgG), as shown in Fig. 1d. These results showed that 10 μM BA-j can inhibit the expression of CDC25C, CDK1 (p-cdc2-Tyr15-Thr14), CDK1 (p-cdc2-Tyr15) and Cyclin B1, rather than CDK2, CDK4, CDK6, Cyclin D and Cyclin E.

Because BA-j can capture ^·^O_2_^−^ and selectively increase the level of H_2_O_2_ in Hep G2 cells, the activation of CDK1 was inhibited by H_2_O_2_ oxidizing CDC25C. These results suggest that BA-j can inhibit CDK1/cyclin B1 by oxidizing CDC25C, rather than other CDKs.

### Anticancer activity

#### *In vitro* anti-proliferative effects of BA-j on cancer cells

The anti-proliferation activity of the BA Mannich base derivatives was screened, and BA-j was the most potent. BA-j had anti-proliferative effects on 12 human cancer cell lines *in vitro* and its IC_50_ was 12.3 μM (4.7 μg/ml, determined via MTT assay), approximately 4-fold more potent than BA (IC_50_ approximately 50 μM)^11^, as shown in Table 1. The average rate of proliferation inhibition in 60 types of human cancer cells treated with 10 μM BA-j was 40.6% (U.S. National Cancer and AIDS Research Center, by SRB). The SFDA “guidelines for antitumor drug research” provide that when the IC_50_ values of compounds or natural drugs inhibiting cancer cell proliferation are less than 10 μg/ml or 20 μg/ml, respectively, they are considered as having anticancer activity *in vitro*. When the tumor inhibitory rate is higher than 40%, the drug is considered to have antitumor activity *in vivo*. Our results indicate that BA-j has significant, broad-spectrum anti-proliferation activity in cancer, with no selectivity for a specific type of cancer. Furthermore, unlike cytotoxic anticancer drugs, BA-j can even reduce the growth of drug-resistant cancer cells.

#### BA-j selectively inhibits proliferation in cancer cells rather than normal cells

**Figure 2:**
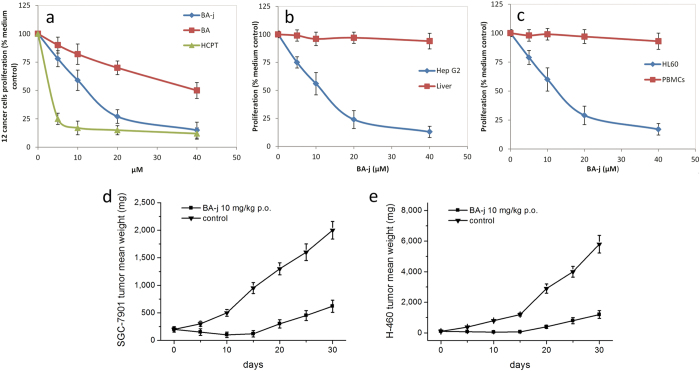
BA-j Anti-proliferation Activity in human cancer cells and 2 tumor xenografts. Cells were cultured in the presence of BA-j for 48 h and an MTT assay was used to determine the anti-proliferation activity. (**a**) Anti-proliferation activity of BA-j in 12 human cancer cell lines compared with BA and HCPT treatment. (**b**) BA-j anti-proliferation activity comparison between Hep G2 cells and normal Liver cells. (**c**) BA-j anti-proliferation activity comparison between MCF-7 cells and normal PBMCs. (**d,e**) Tumor xenograft models of SGC-7901 and H-460 cells in male BALB/c nude mice treated with p.o. 10 mg/kg BA-j twice daily for 10 days.

To examine the effects of BA-j on normal cells, normal human primary hepatocyte cells (Liver) and normal human peripheral blood mononuclear cells (PBMCs) were cultured in the presence of BA-j for 48 h. The anti-proliferative activity of BA-j in normal cells was compared with that in Hep G2 and HL-60 cancer cells, shown in Fig. 2a–c. These results showed that BA-j exclusively inhibited proliferation in Hep G2 and HL-60 cells rather than normal Liver cells or PBMCs. These results indicate that BA-j has anti-proliferative activity in cancer cells rather than to normal cells, while the cytotoxic drug HCPT was active in both cancer cells and normal cells.

In addition, when using BA-j with Acetyi-cysteine (as H_2_O_2_ scavenger) as combined treatment to Hep G2 and HL-60, the anti-proliferation activity of BA-j was inhibited determined by MTT, and the level of H_2_O_2_ reduced measured by PF1. Because BA-j can capture ^·^O_2_^−^ and release H_2_O_2_ with oxidizing Acetyl-cysteine into cystine, BA-j inducing apoptosis was inhibited by Acetyl-cysteine. These results showed that Acetyl-cysteine can relieve the anti-proliferation ability of BA-j in cancer cells by reducing H_2_O_2_^46^. (See Table 2) Thus it is suggested that CDC25C can be oxidized by H_2_O_2_ that is released by BA-j capturing ^·^O_2_^−^, resulting in anti-proliferation eventually.

#### *In vivo* anti-tumor efficacy of BA-j

The antitumor efficacy of BA-j in xenograft models of the human gastric carcinoma (SGC-7901) and non–small cell lung carcinoma (H-460) in BALB/c nude mice was further evaluated. Oral administration of 10 mg/kg BA-j twice daily for 10 days resulted in a significant reduction in tumor growth in mice bearing SGC-7901 and H-460 xenografts and an average weight loss of 10–15% compared with the control group. After 10 days of BA-j treatment, the tumor volume was decreased by >50% of the original tumor size in mice bearing SGC-7901 and H-460 xenografts (P < 0.05). These results indicate that the administration of BA-j can reduce the volume of SGC-7901 and H-460 tumors. The growth inhibition in mice bearing SGC-7901 and H-460 tumors on the 30th day after randomization was 76.7 ± 8.2% and 82.1 ± 10.3%, respectively, compared with the control group (n = 9, P < 0.01), as shown in Fig. 2d,e. These results indicate that BA-j (10 mg/kg) can reduce the growth of SGC-7901 and H-460 xenografts *in vivo* by >75%.

LD_50_ of injection administration of BA-j in mice is 1g/kg. To examine the effects of BA-j on normal mice, oral administration of 10 mg/kg BA-j twice daily for 10 days was conducted, and it showed almost no toxicity or abnormality by tissue sections. These results indicated that BA-j had no effects on normal mice.

### Molecular mechanism of inducing apoptosis in cancer cells

#### Morphological observation of apoptosis induction in MCF-7 cells

**Figure 3:**
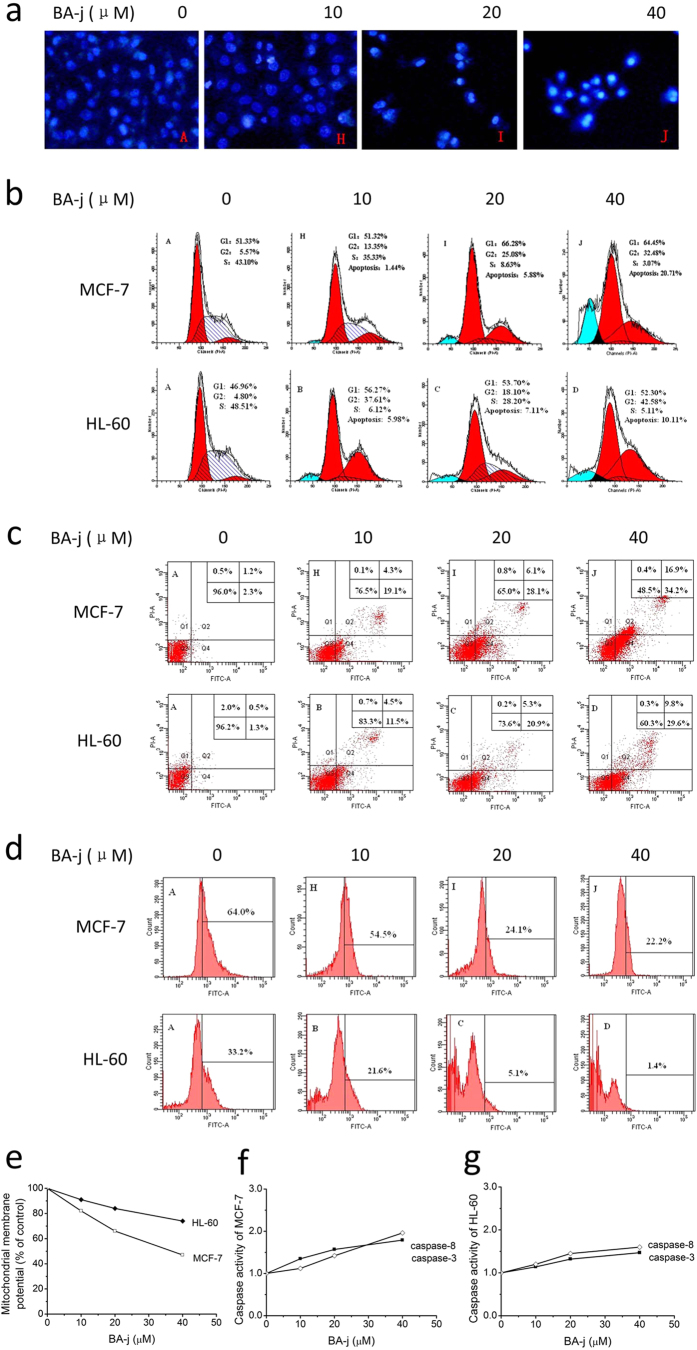
Apoptosis Effects Assays of BA-j. (**a**) Fluorescent images of Hoechst 33258 stained MCF-7 cells showing the induction of apoptosis following a 48 h BA-j treatment (200×). (**b**) Effects of BA-j treatment (48 h) on the cell cycle in MCF-7 and HL-60 cells were determined using PI staining. (**c**) Treating MCF-7 and HL-60 for 48 h with BA-j induces apoptosis. Cell apoptosis was demonstrated using the Annexin V-FITC and PI method. (**d**) Inhibition of Bcl-2 expression after a 48 h BA-j treatment in MCF-7 and HL-60 cells. (**e**–**g**) The MMP is decreased and caspases are activated in MCF-7 and HL-60 cells treated with BA-j for 48 h. (**e**) BA-j treatment decreased the MMP in MCF-7 and HL-60 cells. (**f**) BA-j treatment activated caspase-8 and caspase-3 in MCF-7 cells. (**g**) BA-j treatment activated caspase-8 and caspase-3 in HL-60 cells.

Hoechst 33258 is an active fluorochrome that specifically binds DNA, mainly at A-T rich areas, emitting a blue fluorescence under UV excitation. The nuclei of living cells emit a uniform fluorescence, while apoptotic cells emit a bright blue fluorescence because of chromatin condensation and dense staining of the compact nuclei. After being treated with BA-j for 48 h, MCF-7 cells were stained by Hoechst 33258. Effects on the cellular morphology of these cells are shown in Fig. 3a. Increasing level of BA-j resulted in a sharp decrease in the number of living cells, as well as dense fluorescence emission from nuclei and the appearance of apoptotic bodies around the karyotheca margin, in a remarkably dose-dependent manner. These results indicate that BA-j can significantly induce apoptosis in MCF-7 breast cancer cells.

#### Effects on the cell cycle

PI can specifically bind to DNA, with a good linear relationship between the fluorescence intensity and binding. After being fixed and stained with PI, the DNA content in cells can be quantitatively analyzed using flow cytometry. Because different cell cycle phases have varying levels of DNA content, the effects of a drug on progression through the cell phases can thus being determined. There are many distinct changes at the cellular and molecular level that occur during apoptosis, among the most characteristic of which are changes to the nucleus. Activated endonuclease results in DNA degradation, a decrease in the number of apoptotic cells, and the appearance of a hypodiploid apoptosis peak (sub-G1) before the G0/G1 peak. The results of PI fluorescence staining of MCF-7 and HL-60 cells treated with BA-j for 48 h are shown in Fig. 3b. BA-j treatment led to dose-dependent arrests in the G1 and G2 phase in MCF-7 and HL-60 cells, and a dose-dependent growth in the proportion of the apoptotic peak (sub-G1). Additionally, a high level of the drug resulted in a reduction in the number of cells in S phase. There was no DNA <185 Da in sub-G1. These results indicate that BA-j can induce G1 and G2/M arrest, reduce the number of cells in S phase, and induce apoptosis, rather than inflammation and necrosis, in MCF-7 and HL-60 cells.

#### Apoptosis induction assay

A series of morphological changes occur during apoptosis; changes in the plasma membrane are characteristic of early apoptosis. When cells begin to undergo apoptosis, the cell membrane phospholipid phthalein serine (PS) turns from the inside to the outside of the membrane, becoming exposed on the cell surface. Annexin V is a Ca^2+^-dependent phospholipid-binding protein, which possesses a high affinity to PS and can specifically bind to the PS exposed on the outside of the plasma membrane. However, PS transfers to the outside of the membrane not only during apoptosis but also during cell necrosis. The two mechanisms of death can be distinguished by the fact that the membrane is intact during the initial phases of apoptosis, but broken in cells undergoing necrosis. PI is a dye for nucleic acid that can permeate the membrane and stain the nuclear content in advanced stages of apoptosis and necrosis, but cannot penetrate the intact membrane of living and early-apoptotic cells. Thus, using Annexin V-FITC together with PI, the ratio of apoptotic and necrotic cells can be determined by flow cytometry. The results of co-staining with Annexin V-FITC and PI fluorescent dyes in MCF-7 and HL-60 cells treated with BA-j for 48 h are shown in Fig. 3c. BA-j dose-dependently induced apoptosis in MCF-7 and HL-60 cells. When MCF-7 and HL-60 cells were treated with 40 μM BA-j for 48 h, the percentage of early necrotic cells did not change, whereas the percentage of early apoptotic cells increased to 34.2% and 29.6%, respectively, and that of late apoptotic cells to 16.9% and 9.8%; therefore, total apoptotic cells increased to 51.1% and 39.4%. These data show that the amount of early apoptotic cells remarkably increased with higher drug level, while the increase in late apoptotic cells at this level was more modest. In contrast, the cytotoxic anticancer drug HCPT induced apoptosis at a low level, but inflammation and necrosis at a high level. These results suggest that unlike cytotoxic anticancer drugs, the anti-proliferative activity of BA-j in MCF-7 and HL-60 cells is associated with exclusively inducing apoptosis rather than directly killing cells or causing cell inflammation and necrosis.

#### BA-j decreases the expression of Bcl-2

The endoplasmic reticulum plays an important role in cell apoptosis; the anti-apoptotic protein Bcl-2 and the pro-apoptotic protein Bax reside on it. The balance of Bcl-2/Bax regulates the promotion and inhibition of cell apoptosis. Inhibiting the expression of Bcl-2 can result in the activation of caspases, leading to a cascade reaction, ultimately resulting in cell apoptosis. The expression levels of Bcl-2 in MCF-7 and HL-60 cells treated with BA-j for 48 h are shown in Fig. 3d. The expression of Bcl-2 showed a dose-dependent decrease following BA-j treatment, while there was no difference in Bax expression. These results suggest that BA-j inhibits the expression of Bcl-2, and this inhibition is associated with activation of the intracellular endoplasmic reticulum apoptosis pathway.

#### BA-j decreases the mitochondrial membrane potential

Mitochondria play a key role in cell apoptosis. The asymmetric distribution of protons and other ions on either side of the mitochondrial membrane creates the mitochondrial membrane potential (MMP). Decreasing the MMP are results in the opening of mitochondria permeability transition pores, and the release of pro-apoptotic active substances, such as cytochrome C, from the mitochondrial matrix into the cytoplasm. This results in the activation of caspase-9, which is the start of the mitochondrial apoptosis pathway, and causes a cascade reaction leading to further activation of caspase-3, ultimately resulting in cell apoptosis. Rhodamine 123 is an MMP indicator that can enter the mitochondrial matrix of living cells depending on the current MMP. When Rhodamine 123 is in the mitochondrial matrix, its fluorescence is diminished. During apoptosis, the integrity of the membrane is undermined and mitochondria permeability transition pores open, causing a collapse of the MMP and the release of Rhodamine 123 from mitochondria. This results in a strong, yellow-green fluorescence, whose intensity can be used to determine changes in the MMP. MCF-7 and HL-60 cells were treated with BA-j for 48 h and their Rhodamine123 fluorescence staining results are shown in Fig. 3e. The MMP was decreased with BA-j treatment in a dose-dependent manner. These results suggest that BA-j decreases the MMP, and this decrease is associated with activation of the intracellular mitochondria apoptosis pathway.

#### Activation of caspase-3 and caspase-8

Caspases (cysteine aspartic specific proteases) are a family of aspartic proteases containing cysteine, which possess similar structures and exist in the cytoplasm. Caspases are closely linked to apoptosis and are also involved in the regulation of cell growth and differentiation. Caspases are key enzymes in the process of apoptosis. Caspases are involved in almost all of the signal transduction pathways that mediate apoptosis. Caspases 8, 9, and 12 are early stage initiators of cell apoptosis, and caspases 3, 6, and 7 are late-stage executors. The active site of the caspases is a sulfydryl of cysteine, which exists in the inactive pro-caspase state and is sensitive to H_2_O_2_. When a pro-caspase is oxidized by H_2_O_2_ into a -S-S- dimer, it transforms into an active caspase-S-S-caspase, thus inducing apoptosis. If the sulfydryl of pro-caspase-9 is free, it will combine with phosphorylated survivin, which inhibits apoptosis and promotes cell division. Conversely, if the sulfydryl of pro-caspase-9 is oxidized by H_2_O_2_, it will separate from the phosphorylated survivin, which inhibits cell division and promotes apoptosis. MCF-7 and HL-60 cells were treated with BA-j for 48 h, and the activities of caspase-3 and caspase-8 were determined, as shown in Fig. 3f,g. The activity of caspase-3 and caspase-8 increased in a dose-dependent manner. These results indicate that the induction of apoptosis by BA-j in MCF-7 and HL-60 cells is associated with the activation of caspase-8 and caspase-3, via direct H_2_O_2_ oxidation, rather than death receptor-mediation.

#### BA-j inactivates Fas/FasL

Death receptors are transmembrane proteins that have numerous extracellular cysteine residues and possess intracellular proteolytic activity that further transmits the death signal. Death receptors belong to the tumor necrosis factor receptor family that includes Fas/FasL, TNFR-1/TNFR-α and TRAILR/TRAIL. Tumor necrosis factor receptors cannot be oxidized and activated by ^·^O_2_^−^ or H_2_O_2_, but can be oxidized and activated by NaOCl, which has a stronger oxidation power. Cytotoxic agents can stimulate mitochondria to release NaOCl and activate death factor receptors, thus causing cell inflammation and necrosis.

**Figure 4:**
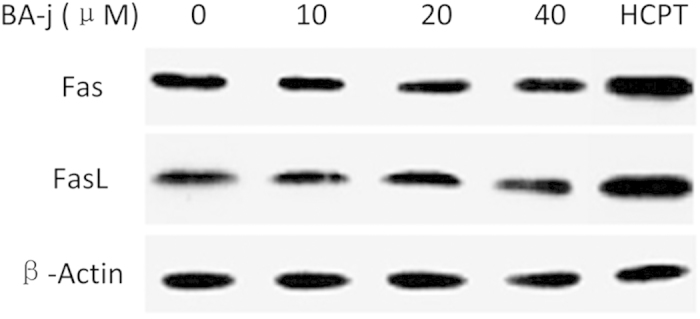
Expression of Fas/FasL in Hep G2 cells treated with BA-j for 48 h compared with HCPT treatment. 2 μM HCPT was used.

Hep G2 cells were treated with BA-j for 48 h, and the expression of Fas/FasL was determined. As shown in Fig. 4, there was no dose-dependent effect of BA-j on Fas levels, whereas 2 μM of HCPT, a cytotoxic anticancer drug used as a positive control, showed an obvious induction of Fas/FasL expression.

These results suggest that the cell inflammation and necrosis caused by cytotoxic anticancer drugs is closely associated with extrinsic death receptors, which results in damage to both cancer cells and normal cells. These data also suggest that BA-j’s bypassing of the extrinsic death receptor pathway is associated with its resistance to NaOCl oxidation, which allows BA-j to protect normal cells from inflammation and necrosis.

### Biochemical mechanism of inducing apoptosis in cancer cells by regulating ROS

#### Resistance to NaOCl oxidation is essential for protecting cells from inflammation and necrosis

BA-j (E^0^ = 0.80 ev) can be oxidized into 8-hydroxymethyl-5, 6-dehydrogenation-baicalein by capturing ^·^O_2_^−^ and releasing H_2_O_2_ (determined by PF1 oxidizing into fluorescein), but BA-j cannot be oxidized by H_2_O_2_. BA-j can also be oxidized into 8-hydroxymethyl-5, 6-dehydrogenation-baicalein by NaOCl without releasing H_2_O_2_ (thus, PF1 is not oxidized into fluorescein), hydrogenating NaOCl into NaCl and H_2_O. ^·^O_2_^−^ has a short survival, and it cannot directly oxidize a sulfhydryl of cysteine. H_2_O_2_ (E^0^ = 0.68 ev) can oxidize a sulfhydryl of cysteine into a disulfide bond rather than a sulfur-oxygen group. NaOCl (E^0^ = 1.49 ev) can oxidize a sulfhydryl of cysteine into both disulfide bonds and sulfur-oxygen groups. These results suggest that the resistance of BA-j to NaOCl oxidation is associated with protecting cells from inflammation and necrosis.

#### Increased intracellular H_2_O_2_ level is essential for selectively inducing apoptosis in cancer cells

**Figure 5:**
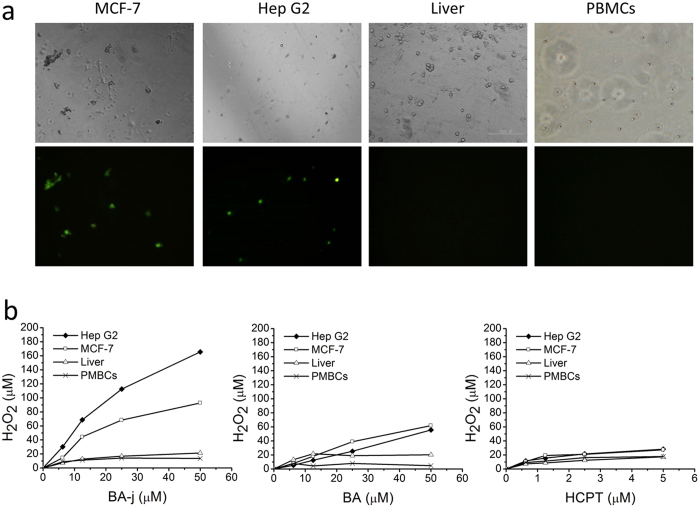
Comparison of BA-j effects on cancer cells and normal cells. (**a**) Fluorescent imaging of intracellular H_2_O_2_ (detected by PF1) in Hep G2 and MCF-7 cancer cells compared with normal Liver cells and PBMCs. All cells were treated with 12.5 μM BA-j for 4 h. (Top: bright field, Bottom: excitation wavelength 488 nm, emission wavelength 525 nm, 64×). (**b**) Assay of intracellular H_2_O_2_ following drug treatment in Hep G2 and MCF-7 cancer cells compared with normal Liver cells and PBMCs. The cells were cultured in the presence of drug for 4 h and the level of intracellular H_2_O_2_ was detected by PF1.

We established a method of measuring the intracellular H_2_O_2_ level using PF1 selective fluorescent probes. To investigate the mechanism of the selective induction of apoptosis in cancer cells and the regulation of intracellular reactive oxygen species (ROS) by BA-j, an equal amount of primary human hepatocytes (Liver) and human peripheral blood mononuclear cells (PBMCs) were prepared and treated with BA-j for 48 h and compared with Hep G2 and MCF-7 cancer cells in intracellular H_2_O_2_ tests (PF1 method). Fig. 5a shows the fluorescence in Hep G2 and MCF-7 cancer cells treated with 12.5 μM BA-j for 4 h. Treating cells with a lower level of BA-j resulted in a weaker fluorescent emission, whereas higher levels of BA-j made most of the cells become apoptotic and increased the number of fluorescent bodies.

Hep G2, Liver, MCF-7 and PBMCs were incubated with BA-j, BA, or HCPT for 4 h and the level of H_2_O_2_ was detected using PF1 fluorescence probes, as shown in Fig. 5b. When Hep G2 and MCF-7 cancer cells were incubated with BA-j, H_2_O_2_ levels exhibited a remarkable dose-dependent increase, whereas Liver cells and PBMCs did not. BA-j can selectively increase the H_2_O_2_ level in Hep G2 and MCF-7 cancer cells rather than in normal Liver cells and PBMCs by capturing ^·^O_2_^−^ and releasing H_2_O_2_. BA-j is also associated with selectively inducing apoptosis in cancer cells but not in normal cells. By contrast, the results indicated that the cytotoxic drug HCPT had no selectivity in increasing H_2_O_2_ levels; it would harm normal cells and kill cancer cells. BA-j also was resistant to NaOCl oxidation, which is associated with preventing inflammation and necrosis in cells.

#### Metabolic pathway capturing ^·^O_2_^−^ and releasing H_2_O_2_

**Figure 6:**
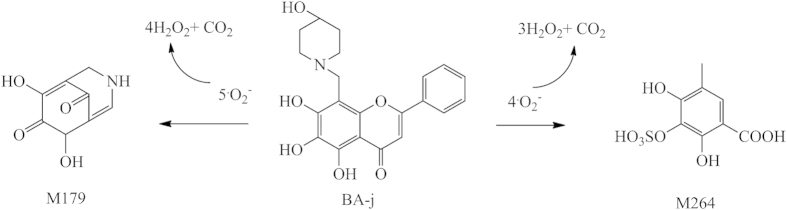
The major metabolic pathway of BA-j in macaques.

The major metabolic pathway of BA-j in macaques was identified by LC-MS-MS. The major metabolic pathway involved BA-j capturing 4-5 molecules of ^·^O_2_^−^ and releasing 3-4 molecules of H_2_O_2_ based on the data given in Fig. 5b, with itself degrading into active intermediate metabolite dihydroflavonol, then while being oxidized and degraded mainly into the metabolites M179 and M264, as shown in Fig. 6. 95% of the BA that entered the body was excreted after glucuronidation or sulfation, and only a few of the BA molecules captured 1 molecule of ^·^O_2_^−^ and released 1 molecule of H_2_O_2_ each, thus becoming oxidized. 4-hydroxylpiperidine possessed a strong activity against oxygen radicals because it captured multi-molecular ^·^O_2_^−^, and released multi-molecular H_2_O_2_ as it became oxidized and degraded. Introduction of 4-hydroxylpiperidine methylene into BA-activated flavonoid rings resulted in the easy capture of ^·^O_2_^−^ and self-degradation. Meanwhile, the “enterohepatic efflux effect” by direct glucuronidation and sulfation was obviously inhibited. These results suggest that BA-j captures ^·^O_2_^−^ *in vivo*, is degraded into M179 and M264, and releases H_2_O_2_, which is associated with increasing the intracellular H_2_O_2_ level in cancer cells. This metabolic pathway can inhibit the BA-j “efflux effect” caused by direct glucuronidation and sulfation. The pharmacokinetic nature of BA-j was better than that of BA.

## Discussion

David Santamarıá *et al.* indicated in his 2007 article in Nature that CDK1 is the only essential CDK in cell proliferation. Moreover, in the absence of interphase CDKs, CDK1 can execute all the events that are required to drive cell^1^. When CDK1 is phosphorylated into CDK1 (p-cdc2-Tyr15-Thr14) by Weel and Mytl protein kinase, CDC25C dephosphorylate it into active CDK1 (p-cdc2-Tyr15), which combines Cyclin B1 and phosphorylate the substrate Survivin into Survivin (p-Thr14), thus promoting the cells into mitosis phase^47^. In 2010, Jorrit M Enserink and Richard D Kolodner posed that CDK1 could be used as the latest target for anti-proliferation drug research^48^.

BA is known to have selective inhibitory ability against CDK1, and inhibitory activity is weak against other kinases^32^. We found that BA-j as a substrate compound for ATP can directly inhibit the activity of CDK1 studied by FRET which is the enzyme activity determination method of the CDK1 in non-cancer cells, and the inhibitory activity of BA-j (CC_50_ 0.30 μM) against CDK1/Cyclin B1 is approximately 20-fold and 50-fold stronger than BA and Baicalin. Because 4-hydroxy-piperidin as a potent antioxidant can capture multi-molecules of ^·^O_2_^−^ and release multi-molecules of H_2_O_2_, BA was structurally modified into BA-j by Mannich with 4-hydroxy-piperidin, and the druggbility of BA-j is more excellent. It is worth noting that the former is the activity determination result of the CDK1 enzyme of non-cancer cells using FRET method, while the latter is the determination result in cancer cells *in vitro* by MTT. Principles are different between these two methods. Therefore, the results can only be compared qualitatively, but not quantitatively.

In Hep G2 cells, we founded that BA-j can selectively inhibit CDK1/Cyclin B1, rather than CDK2/Cyclin E or CDK4, 6/Cyclin D. BA-j can inhibit CDC25C too with dephosphorylating CDK1 (p-cdc2-Tyr15-Thr14) into active CDK1 (p-cdc2-Tyr15). Because BA-j can capture ^·^O_2_^−^ and selectively increase the level of H_2_O_2_ in cancer cells rather than normal cells, which can oxidize CDC25C, and the dephosphorylation of CDK1is inhibited, but not other CDKs, thus inhibiting cell proliferation.

These results suggest that BA-j as a substrate compound for ATP is not only a direct inhibitor of CDK1/cyclin B, but also an indirect inhibitor of CDK1/cyclin B1 by H_2_O_2_ oxidizing CDC25C rather than of other CDKs. Therefore, BA-j is a novel selective CDK1 inhibitor.

BA-j showed broad-spectrum anti-cancer activity (IC_50_ 12.3 μM, 4.7 μg/ml) and approximately 4-fold stronger than BA (IC_50_ 50 μM) measured by MTT^11^. The anti proliferative activity of BA and Baicalin is not significant *in vivo*, because of their poor bioavailability by “enterohepatic efflux effects”. Because druggbility of BA-j is more excellent than BA, BA-j can significantly reduce the growth of SGC-7901 and H-460 xenografts (10 mg/kg, 10 day, >75%). The SFDA “guidelines for antitumor drug research” provide that when the IC_50_ values of compounds or natural drugs inhibiting cancer cell proliferation are less 10 μg/ml or 20 μg/ml, respectively, they are considered as having anticancer activity *in vitro*. When the tumor growth inhibitory rate is more than 40%, a drug is considered as having significant anti-tumor activity *in vivo*. BA-j showed no significant side effects within the therapeutic dosage range. Accordingly, the data suggest that BA-j has significant anti-tumor activity with high effect and lower toxicity. ROS are important messengers in cell signaling processes and form the biochemical basis of many physiological and pathological responses. ROS include oxygen free radicals (^·^O_2_^−^), hydroxyl radicals (^·^OH^−^), hydrogen peroxide (H_2_O_2_), sodium hypochlorite (NaOCl), nitric oxide (NO), lipid peroxides (ROOH), over oxygen nitrite (^·^ONOO^−^) and so on. H_2_O_2_ has a high diffusivity and can pass through the membrane. High levels of H_2_O_2_ can oxidize NaCl into NaOCl by catalysis with catalase (CAT).

The mechanisms of redox states are different between normal cells and cancer cells. In normal cells, where the activity of superoxide dismutase (SOD) is relatively high, over 95% of the ^·^O_2_^−^ generated can be captured and composed into H_2_O_2_ by SOD. Then, excessive H_2_O_2_ is catalyzed and decomposed into H_2_O and O_2_ by glutathione enzyme (GPX), which oxidizes glutathione (GSH) into oxidized glutathione (GSSG); thus SOD and GPX maintain relatively low levels of ^·^O_2_^−^ and H_2_O_2_, and the cells remain in a non-proliferative state. Whereas in tumor cells, the activity of SOD and CAT is weaker than in normal cells, leading to the accumulation of ^·^O_2_^−^, which promotes cell proliferation and increases the sensitivity of these cells to H_2_O_2_. Compared with normal cells, tumor cells are more likely to be induced to undergo apoptosis by the same level of H_2_O_2_.

Carole Nicco’s research group from Faculté de Médecine, University Paris V demonstrated for the first time that among the antioxidant enzymes that detoxify H_2_O_2_, the glutathione pathway is only weakly involved in the control of H_2_O_2_ production by tumor cells, but plays a major role in the control of H_2_O_2_ production in non-transformed cells. Thus, in normal cells, ROS are kept at low levels because the activity of NADPH oxidase and the H_2_O_2_ levels are regulated by the glutathione system. By contrast, in tumor cells, high levels of ROS, close to the threshold of cytotoxicity, are produced through the mitochondrial respiratory chain, and the H_2_O_2_ level is controlled by CAT. This discovery suggested for the first time that any agent that increases intracellular H_2_O_2_ levels could slow down cancer proliferation and lead to cell apoptosis. Conversely, any agent that decreases the intracellular H_2_O_2_ level could enhance tumor growth. H_2_O_2_ plays a pivotal role in controlling the fate of normal and tumor cells. Therefore, drugs that specifically increase intracellular H_2_O_2_ level in cancer cells can selectively induce cancer apoptosis^46^.

The effects of ROS on cell proliferation depend on their nature, sub-cellular origin and intracellular level. The intracellular level of H_2_O_2_ is critical because it easily reaches the threshold of toxicity in tumor cells. When some stimulation causes the H_2_O_2_ level in tumor cells to climb high enough, kinases with their active site of cysteine can become oxidized, for instance, H_2_O_2_ can cause depletion of the GSH content in cancer cells, target oxidation of CDC25, and inhibit activation of CDKs/Cyclins, suppressing proliferation^47^. H_2_O_2_ also can specifically oxidize caspases, thus activating the apoptotic pathway of mitochondria and endoplasmic reticulum and selectively inducing apoptosis in tumor cells. At low levels, cytotoxic drugs can improve the intracellular H_2_O_2_ level in cancer cells, oxidizing and activating the intrinsic apoptosis pathway. However, at high levels, they can stimulate mitochondria to produce NaOCl by H_2_O_2_, oxidizing NaCl with CAT catalysis, thus activating the extrinsic death receptors pathway and causing cell inflammation and necrosis. Therefore, cytotoxic drugs damage normal cells while killing cancer cells^49^.

The molecular mechanism responsible for the selectivity of BA-j is a result of the differential regulation of ROS between normal cells and cancer cells, which allows BA-j to selectively induce apoptosis in cancer cells while protecting normal cells from inflammation and necrosis. In normal cells, the activity of SOD and GPX is high, and ^·^O_2_^−^ levels are low. BA-j captures ^·^O_2_^−^ and produces small amounts of H_2_O_2_ that can be maintained at a very low level by GPX control, so the cells remain in a non-proliferative state. Whereas in cancer cells which have lower activity of SOD and CAT than normal cells, ^·^O_2_^−^ is kept at a higher level. BA-j, similar to SOD, can capture ^·^O_2_^−^ but produces more amounts of H_2_O_2_, which is maintained at a higher level.

The higher level of H_2_O_2_ can specifically oxidize kinases with active site of cysteine. For example, in cancer cells, H_2_O_2_ can cause depletion of GSH content^50^, target oxidation of CDC25, and inactivate CDKs/Cyclins, thus suppressing cancer proliferation^15^,^19^,^25^,^32^,^47^. H_2_O_2_ can specifically oxidize and activate the intrinsic apoptosis pathway (i.e., reduce the mitochondrial membrane potential, inhibit expression of Bcl-2, and activate caspase-8 and caspase-3, and therefore inducing apoptosis in cancer cells.

BA is known to have ROS-generating ability. However, the exact biochemical mechanism of BA regulating ROS is not understood. Because of their using of DCFH-DA, a non selective a probe, only the total ROS in cells was determined, but the differences among ^·^O_2_^−^, H_2_O_2_ and NaOCl were not distinguished. Therefore, questions such as why BA is different from cytotoxic anti-cancer drugs which harm normal cells while killing cancer cells could not be explained^33^,^51^.

**Figure 7:**
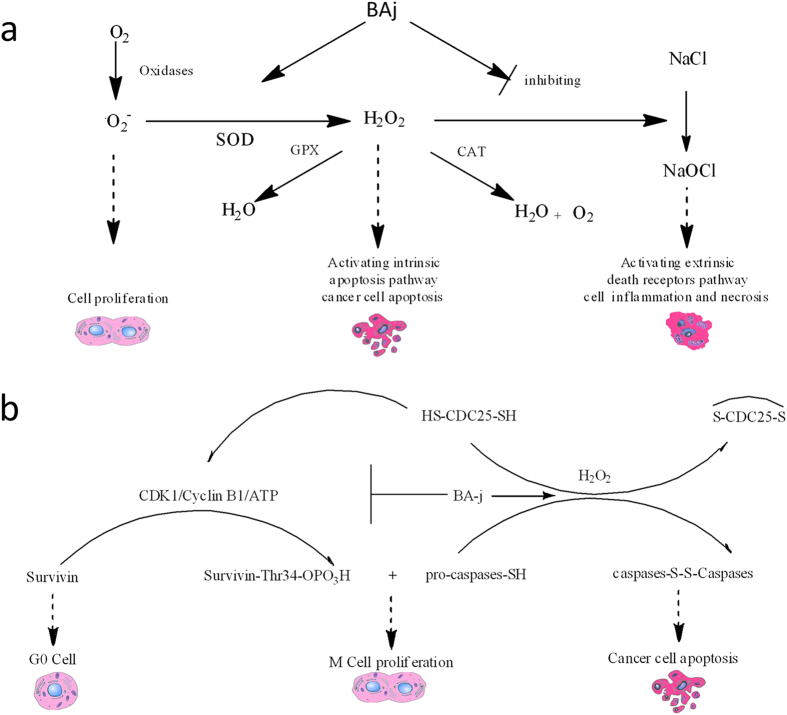
Mechanisms of ROS regulations. (**a**) Molecular mechanisms of H_2_O_2_–regulated cell proliferation and apoptosis. (**b**) Mechanisms of ROS regulation by BA-j. BA-j can selectively induce apoptosis in cancer cells and protect normal cells from inflammation and necrosis.

In this paper, a novel method for assaying intracellular H_2_O_2_ was designed by using the H_2_O_2_ selective fluorescent probe PF1^52^. We First confirmed that BA-j can regulate ROS level (reducing ^·^O_2_^−^, increasing H_2_O_2_, reducing NaOCl), but cytotoxic anti-cancer drugs can increase the ROS level (increasing ^·^O_2_^−^, H_2_O_2_, and NaOCl). Because of the differential mechanisms controlling redox states in normal and cancer cells, BA-j can capture oxygen free radicals (^·^O_2_^−^) and selectively increase the level of H_2_O_2_ in cancer cells, thereby specifically oxidize some enzymes with active site of cysteine, specifically oxidize and activate the intrinsic apoptosis pathway. BA-j can also resist oxidation by NaOCl, bypassing the extrinsic death receptor pathway (i.e., inactivate tumor necrosis factor receptor Fas/Fasl), which allows it to protect cells from inflammation and necrosis^16^,^31^. BA-j showed a higher activity but similar molecular mechanism to BA; see Fig. 7. This mechanism of BA-j is different from cytotoxic anticancer drugs, which can activate both the intrinsic apoptosis pathway and the extrinsic death receptors pathway, and therefore harm normal cells while killing cancer cells.

## Conclusion

The molecular and biochemical mechanisms of BA-j is the direct inhibition of CDK1 and capturing ^·^O_2_^−^ and increasing level of H_2_O_2_ in cancer cells rather than in normal cells. Higher level of H_2_O_2_ is capable of specifically oxidizing kinases with cysteine at their active sites, such as oxidizing CDC25 with inactivating CDK1, and thus inhibiting proliferation cells, and oxidizing Caspases and activating the intrinsic apoptosis pathway thus inducing apoptosis in cancer cells rather than in normal cells. BA-j can also resist NaOCl oxidation, bypassing the extrinsic death receptor pathway, which allows it to protect cells from inflammation and necrosis. BA-j has broad-spectrum anticancer activity and can significantly reduce the growth of SGC-7901 and H-460 xenografts. These data provide evidence that BA-j may be developed into a novel anticancer drug.

## Materials and Methods

### Ethical Statements

Animal experiments were conducted in accordance with the guidelines issued by the State Food and Drug Administration (SFDA of China). The research and protocol were approved by the Animal Care and Use Committee of Dalian Medical University in China. The animals were housed and cared for in accordance with the guidelines established by the National Science Council of Republic China. The present study was performed in accordance with the Guide for the Care and Use of Laboratory Animals (National Research Council, NIH Publication No. 85–23, 1996). Briefly, male (6–8 week-old, 18–22 g, mixed-sex) BALB/c nude mice were provided by the Animal Care and Use Committee of Dalian Medical University in China.

### Assay of intracellular H_2_O_2_ by PF1

PF1 was designed as an intracellular H_2_O_2_ selective fluorescent probe by Christopher J. Chang from the University of California-Berkeley. This probe is permeable through the cell membrane, stable and selective. PF1 is not fluorescent on its own, but it can be oxidized into yellow-green fluorescein FITC selectively by intracellular H_2_O_2_, rather than by ^·^O_2_^−^ or NaOCl. PF1 is currently the only truly effective tool for assessing intracellular H_2_O_2_ levels induced by a drug^52^. A novel method to assay intracellular H_2_O_2_ was designed using the selective H_2_O_2_ fluorescent probe PF1 (synthesized by us), which was better than the non-selective ROS fluorescent probe DCFH-DA (2′, 7′-dichlorofluorescin diacetate)^49^.

Determination method: cancer cells were seeded in a 96-well black-transparent micro-plate (1 × 10^5^ cells/ml and 100 μl/well) and incubated at 37 °C in an atmosphere of 5% CO_2_ for 30 h, or for 6 h in the case of normal cells. Then, 10 μl of a PBS-drug solution (drug was prepared as 4 mg/ml in DMSO and diluted with PBS to 500, 250, 125, 62.5, 31.25 and 0 μM) was added to 4 replicates. Cells were incubated for 3.5 h, then 10 μl of a PF1 fluorescent probes solution was added (a stock solution of PF1 was prepared as 1 mg/ml in DMSO and diluted with PBS to a 100 μM working solution), and cells were further incubated at 37 °C for 0.5 h while the micro-plate was shielded by foil. The fluorescence intensity was detected using fluorescence ELISA (excitation wavelength 488 nm, emission wavelength 525 nm), and the H_2_O_2_ concentration was calculated. For adherent cells, the foil and the media were removed, and the cells were washed with 200 μl PBS before being subjected to intracellular H_2_O_2_ fluorescence imaging via the fluorescence microscope. For suspended cells, 200 μl PBS was added to the media, and after 5 min, cells from the bottom of each well were collected and plated on glass coverslips and subjected to intracellular H_2_O_2_ fluorescence imaging via the fluorescence microscope.

Standard curve and quantitative limits: 90 μl of plasma culture media was added to each well, and then 10 μl of a PBS-H_2_O_2_ solution (H_2_O_2_ solution was diluted with PBS to 500, 250, 125, 62.5, 31.25 and 0 μM) was added to 4 replicates. Next, 10 μl of a 100 μM PF1 fluorescence probes solution was added, and micro-plates were shaken for 0.5 h at 37 °C. Then, the fluorescence intensity was measured and the standard curve was drawn.

The concentration of H_2_O_2_ in the plasma media ranged from 3 to 100 μM, and each well was treated with 10 μM PF1. With the square root of the blank correcting fluorescence OD differences as the abscissa (X), and the concentration of H_2_O_2_ (μM) as the ordinate (Y), Linear regression equation: Y = 0.592X (R^2^ = 0.995, n = 6) was derived, and a good linear relationship was observed.

### CDK1/Cyclin B1 inhibition assay by FRET

The quantitative analysis technique of Fluorescence Resonance Energy Transfer (FRET) was adopted for screening the CDK1/Cyclin B inhibitory activity of the flavonoid Mannich base derivatives. Generally, the test compounds were prepared as 1 mg/ml DMSO solutions and diluted with DMSO into 8 geometric concentrations (3-fold). CDK1/Cyclin B inhibitory activities were measured by a fluorescence kinetic assay and CC_50_ values were calculated according to the instructions of the CDK1/Cyclin B kit (Invitrogen, USA)^44^.

### Cell culture

Breast (MCF-7 [TCHu 74] and MDA-MB-231 [TCHu104]), cervical (Hela [TCHu187]), hepatoma (SMMC-7721 [TCHu 52] and Hep G2 [TCHu 72]), gastric (SGC-7901 [TCHu 46] and BGC-823 [TCHu 11]), prostate (PC-3 [TCHu158]), leukemia (HL-60 [TCHu 23] and K562 [TCHu191]), non–small cell lung carcinoma (H-460 [TCHu205]), and colon (HCT-116 [TCHu 99]) cancer cell lines were purchased from the Cell Bank of Shanghai Institute of Biochemistry and Cell Biology, Chinese Academy of Sciences (Shanghai, China). The catalogue number of each cell line is indicated in the bracket following its name.

The cell lines were seeded in 4 × 6 cm flasks and cultured in DMEM (Sigma) supplemented with 10% heat-inactivated fetal bovine serum (Gibco), 100 U/ml Penicillin and 100 μg/ml streptomycin in a water-saturated atmosphere of 5% CO_2_ at 37 °C. The culture medium was changed every 48 h and cells were sub-cultured approximately once every 96 h. For sub-culturing, the medium was discarded, and the appropriate amount of 0.25% trypsin was added for a 2–3 min digestion at 37 °C. Observed under the microscope, these cells tended to become round. 10% FBS was added to terminate the digestion, and the cell solution was gently beaten with a straw several times so that cells were detached from the culture bottle wall and completely dispersed. These cells were divided into cell culture flasks and the appropriate amount of medium was added for further culture. For HL-60 cells, part of the medium was discarded leaving the appropriate amount of cells and medium, and fresh medium was added. For plating experiments, cells were observed and counted under the microscope, and cells in the logarithmic growth phase were used. RPMI 1640 medium was added for dilution, and cells were counted under the microscope and adjusted to 1 × 10^5^ cells/ml.

Normal human primary hepatocytes (Liver) were purchased from the Dalian Institute of Physical Chemistry (Dalian, China). Liver was cut into small pieces and digested with the appropriate amount of 0.25% trypsin at 37 °C for 2–3 min. Observed under the microscope, these cells tended to become round. 10% FBS was added to terminate the digestion, and the cell solution was gently beaten with a straw several times so that cells were detached from the culture bottle wall and completely dispersed. RPMI 1640 medium was added for dilution, and the cells were counted under the microscope and adjusted to 1 × 10^5^ cells/ml.

Normal human peripheral blood was purchased from the Dalian city blood bank and approved by the human blood use committee of the Dalian city blood bank in China. Normal human peripheral blood mononuclear cells (PBMCs) were isolated from heparinized venous blood of healthy volunteers by Ficoll–Paque density gradient centrifugation. After anticoagulation by heparin, 1 ml of fresh human peripheral blood was washed with PBS twice, and then 1 ml of Ficoll-PM400 was carefully added into the bottom of the test tube, forming a single layer. After 1000 r/min centrifugation for 7 min, the intermediate buffy coat layer was separated. RPMI 1640 medium was added for dilution, and the cells were counted under the microscope and adjusted to 1 × 10^5^ cells/ml.

### Proliferation assay by MTT

Cells were seeded in 96-well micro-plates (5 × 10^3^ cells/well, HL-60 1 × 10^4^ cells/well) and incubated at 37 °C for 24 h. In dark conditions, BA-j-DMSO solutions were diluted with PBS and added to the appropriate wells with final concentrations of 0, 5, 10, 20, and 40 μM, with 4 replicates of each condition. For the control, PBS containing 0.1% DMSO was added instead of the BA-j solution. The micro-plates were incubated at 37 °C for 48 h, and then the media were removed. 100 μl of a 0.5 mg/ml MTT solution diluted with PBS was added to the wells, and the micro-plates were further incubated for 3 h. After incubation, the MTT solution was removed, and 100 μl of DMSO was added to each well to dissolve the formazan. Micro-plates were read at 570 nm (630 nm as a reference) by a micro-plate reader (Sunrise, TECAN, Austria), according to Tim Mosmann’s method.

### Tumor xenograft model

Human gastric carcinoma (SGC-7901) cells were chopped into fragments (approximately 1.5 mm), each of which was transplanted s.c. into the right axillary fossa of BALB/c nude mice. Human non–small carcinoma (H-460) cells were grown in RPMI 1640 containing 10% fetal bovine serum and harvested. Cells were resuspended in saline at 2.5 × 10^7^ cells/ml and placed on ice. Severe combined immunodeficient mice were injected with a 0.2 ml cell suspension s.c. on the right flank and observed daily for tumor appearance. When the tumors attained a diameter of 5 mm, tumor xenograft models were randomized into two groups (n = 9). For 10 days, a 10 mg/kg BA-j mesylate injection was orally administered twice daily in the test group, and water was administered in the control group. Animals in both models were observed daily for signs of health deterioration and mortality for 30 days. Tumor size was measured once every 5 days in two perpendicular dimensions with vernier calipers and converted to tumor volume (TV) using the formula: (ab^2^)/2, where a and b refer to the longer and shorter dimensions, respectively. The body weight of the animals was measured once every 5 days, concurrently with the tumor dimension measurements. After observation for 30 days, mice were euthanized by cervical dislocation (this method of animal sacrifice is in accordance with the criteria set up by the Animal Care and Use Committee of Dalian Medical University in China) and weighed, and the tumor was segregated and weighed. No signs of ill health, pain, or suffering were observed, and therefore no action was taken to minimize suffering. There were no obvious adverse effects or mortality during the experiment.

### Morphological observation of nuclear change

Hoechst 33258 staining method was adopted for analysis under dark conditions. Cells were seeded in 96-well micro-plates (1 × 10^6^ cells/ml, 100 μl/well), and pre-incubated at 37 °C. Then, different concentrations (10, 20 and 40 μM) of BA-j were added, and the micro-plates were further incubated for 48 h. For staining, media were removed and cells were washed with PBS 3 times before being fixed in 0.5 ml of cold, 4% paraformaldehyde for 30 min at room temperature. Then, fixative was removed, and cells were washed with PBS 3 times, stained with 0.5 ml of 10 μg/ml Hoechst 33258 (Sigma) at 37 °C for 10 min, and incubated at 37 °C in 5% CO_2_ for 10 min. The presence of nuclear morphological changes in the cells was observed with an Olympus fluorescence microscope fitted with a UV excitation filter (Tokyo, Japan).

### Cell cycle analysis

Propidium iodide (PI), which binds DNA, was used for staining analysis. Cells were seeded in flasks (5 × 10^4^ cells/ml, 3 ml/flask), and cultured for 24 h in the dark. Then, different concentrations (10, 20 and 40 μM) of BA-j were added, and the micro-plates were further incubated for 48 h. After culturing, cells were collected (approximately 1 × 10^4^), centrifuged at 1000 r/min for 5 min, and washed with cold PBS 3 times. Next, cells were fixed in 70% cold ethanol at 4 °C overnight. Then, fixative was removed, cells were washed with PBS twice, and incubated with DNase-free RNase (100 μg/ml) at 37 °C for 30 min. After incubation, the cells were re-suspended in 10 μg/ml PI (Sigma) solution and incubated at 4 °C for another 30 min in the dark, and then subjected to cell cycle analysis using flow cytometry. Flow cytometry (BD FACS cantoTM, USA) was used to detect cells in different phases of the cell cycle, and the percentages of cells in G1, S, and G2 phases were calculated using ModFit LT 3.0 software.

### Apoptosis assay

Annexin V-FITC antibody immunofluorescence combined with PI/DNA binding was adopted for fluorescent analysis of apoptosis. Cells (2 × 10^5^) were collected and subjected to quantitative flow cytometry analysis according to the instructions of the Annexin V-FITC kit (BioVision, USA).

### Assay of Bcl-2 and Bax expression

Immunofluorescence was used to analyze Bcl-2 and Bax protein expression. Cells (1 × 10^6^) were washed with cold PBS 3 times, fixed with 4% paraformaldehyde at 4 °C for 20 min, and then incubated with 0.5% TritonX-100 and 1% bovine serum albumin for 10 min. The cells were then washed with PBS twice, followed by the addition of primary antibodies against Bcl-2 or Bax (Santa Cruz) and further incubation at 37 °C for 1 h. Next, the cells were washed with PBS twice and incubated with the corresponding fluorescein isothiocyanate (FITC)-conjugated secondary antibodies at room temperature for 45 min in the dark. The cells were washed with PBS again, and the antigen density was analyzed by flow cytometry as described with minor modifications.

### Assay of mitochondrial membrane potential

Mitochondrial membrane potential (MMP) changes were detected and analyzed with the Rhodamine 123 detection kit (Rh123, Nanjing Keygen Biotech., China). Cells (1 × 10^6^) were incubated with 10 μg/ml Rh123 solution for 10 min at 37 °C in an atmosphere of 5% CO_2_. After incubation, the cells were washed twice with PBS to remove free Rh123, and then incubated in PBS for 60 min at 37 °C in an atmosphere of 5% CO_2_. The fluorescence intensity of Rh123 was measured immediately with Thermo Scientific Varioskan Flash (Thermo Scientific, USA) using excitation and emission wavelengths of 500 nm and 530 nm, respectively.

### Caspase activity assay

Because the emission spectrum of free *p*-nitroaniline can be detected by a micro-plate reader at 405 nm, caspase activity can also be determined. A caspase-8 and caspase-3 colorimetric assay kit (Nanjing KeyGen Biotech., China) was used to determine caspase activity. Cells (2 × 10^6^) were collected in a 1.5 ml centrifuge tube and lysed by 10 min incubation with 50 μl lysis buffer on ice. After 1000 r/min centrifugation at 4 °C for 1 min, the supernatant (i.e., cytoplasmic extract) was carefully pipetted into another tube and kept on ice. The protein concentration of each sample was determined using the Bradford method. Then, according to the protein level of each sample, certain amounts of protein (50–200 μg) were diluted in 50 μl cell lysis buffer for the next step of the experiment. Next, 50 μl of 2× reaction buffer (containing 10 mM DTT) and 5 μl of 4 mM DEVD *p*-nitroaniline (final concentration 200 μM) were added, and lysates were incubated at 37 °C for 4 h, then transferred into a 96-well micro-plate and read at 40 nm by a micro-plate reader.

### Western blot analysis

Hep G2 cells (2 × 10^6^) were lysed, and their protein content was separated by SDS-PAGE and electrophoretically transferred onto a PVDF membrane (Millipore). The membrane was blocked with 5% nonfat milk in TBST buffer and incubated overnight at 4 °C with specific primary antibodies, including anti-human Fas and FasL antibodies (Transduction Laboratory, USA). The membrane was washed with TBST buffer and incubated with the appropriate secondary antibody (horseradish peroxidase-conjugated goat-antimouse IgG). Determinations were performed using enhanced chemiluminescence kits (Amersham Biosciences, USA).

The membrane was blocked with 5% nonfat milk in TBST buffer and incubated overnight at 4 °C with specific primary antibodies, including anti-human CDC25C, CDK1 (p-cdc2-Tyr15-Thr14), CDK1 (p-cdc2-Tyr15), CDK2, CDK4, CDK6, Cyclin B1, Cyclin D, Cyclin E antibodies. The membrane was washed with TBST buffer, incubated with the appropriate secondary antibody (goat-antimouse IgG/ FITC), and the membrane was washed with TBST buffer. Differences in protein level were determined for conjugated goat-antimouse IgG/FITC by densitometric scanning (Quantity One software package, Biorad) and normalized using internal standards (i.e., β-actin)^32^.

### Statistical analysis

Data are expressed as the mean ± S.E.M. from 4 repeated experiments and were evaluated using a one-way ANOVA. Differences are considered significant when P < 0.05.

## Additional Information

**How to cite this article**: Zhang, S. *et al.* BA-j as a novel CDK1 inhibitor selectively induces apoptosis in cancer cells by regulating ROS. *Sci. Rep.* **5**, 13626; doi: 10.1038/srep13626 (2015).

## Figures and Tables

**Table 1:** Anti-proliferation IC_50_ of BA-j in 12 cancer cell lines *in vitro* (48 h, determined by MTT assay, n = 4).

No.	Cancer Cell Line	IC_50_ μM	±μM
1	MCF-7	10.6	0.3
2	Hela	8.2	1.54
3	SMMC-7721	11.1	3.02
4	SGC-7901	16.8	1.83
5	HL-60	12.5	1.63
6	BGC-823	7.2	1.73
7	PC-3	12.3	2.06
8	H-460	12.7	2.62
9	Hep G2	10.1	0.30
10	K562	15.5	1.25
11	HCT-116	15.8	3.21
12	MDR-MB-231	14.8	0.84
AV		12.3	1.69

**Table 2:** Acetyl-cysteine inhibiting anti-proliferation and H_2_O_2_ level of BA- j in Hep G2 and HL-60 by MTT(48 h) or by PF1(4 h).

Cancer cells	Hep G2	HL-60	Hep G2	HL-60
BA-j μM	10	10	10	10
Acetyl-cysteine μM	0	0	50	50
Proliferation %	55	60	96	95
H_2_O_2_ μM	40	35	6	5
